# The unresolved definition of the pressure-viscosity coefficient

**DOI:** 10.1038/s41598-022-07470-3

**Published:** 2022-03-01

**Authors:** Scott Bair

**Affiliations:** Georgia Institute of Technology, Center for High-Pressure Rheology, George W. Woodruff School of Mechanical Engineering, Atlanta, GA 30332-0405 USA

**Keywords:** Mechanical engineering, Fluid dynamics

## Abstract

In the classical approach to elastohydrodynamic lubrication (EHL) a single parameter, the pressure-viscosity coefficient, quantifies the isothermal pressure dependence of the viscosity for use in prediction of film thickness. Many definitions are in current use. Progress toward a successful definition of this property has been hampered by the refusal of those working in classical EHL to acknowledge the existence of accurate measurements of the piezoviscous effect that have existed for nearly a century. The Hamrock and Dowson pressure-viscosity coefficient at high temperature requires knowledge of the piezoviscous response at pressures which exceed the inlet pressure and may exceed the Hertz pressure. The definition of pressure-viscosity coefficient and the assumed equation of state must limit the use of the classical formulas, including Hamrock and Dowson, to liquids with high Newtonian limit and to low temperature. Given that this problem has existed for at least fifty years without resolution, it is reasonable to conclude that there is no definition of pressure-viscosity coefficient that will quantify the piezoviscous response for an analytical calculation of EHL film thickness at temperatures above ambient.

## Introduction

Elastohydrodynamic lubrication (EHL) assures that a film of liquid separates the contacting solid machine elements for cams, gears and rolling bearings. Engineers are estimating the thickness of these films with formulas which require quantification of the piezoviscous effect by a pressure-viscosity coefficient. The classical approach to EHL has not employed an accurate description of the pressure dependence of the viscosity of the lubricant, although a comprehensive report on the piezoviscous effect for lubricating oils has been available since 1953^1^ and the methodology for viscosity measurement to 1.2 GPa has existed for nearly a century^2^. Early work in EHL employed a fictional narrative invoking an 1893 article^3^ by a geologist, Carl Barus, on the effective viscosity of extruding solids. Although his pressure-viscosity formula was linear and he specifically rejected the exponential form, stating “marked violence is thus done to the observations”, the relationship given his name in EHL is the exponential form.

1 $$\mu = \mu_{0} \exp \left( {\alpha_{B} p} \right)$$

This is not to say that exponential response does not occur. Real piezoviscous response is slower than exponential (at times even linear) at low pressure and high temperature, becoming faster than exponential at high pressure as the glass transition pressure is approached. Therefore, Eq. (1) can be applied near the inflection in the log viscosity versus pressure curve which may include ambient pressure if the temperature is low. The inflection in the log viscosity versus pressure curve occurs at a characteristic viscosity that is roughly independent of temperature^4^. Here, *μ* is the limiting low shear (Newtonian) viscosity in contrast to *η*, the generalized Newtonian viscosity which varies with shear rate.

One impediment to EHL becoming a quantitative field is the lack of a consensus for the definition of a pressure-viscosity coefficient which sufficiently characterizes the strength of the piezoviscous response in the inlet zone to allow for an accurate prediction of film thickness. A significant hindrance to this goal is the use of unrealistic assumptions in the classical film thickness formulas. Two of the unrealistic assumptions are the Newtonian inlet zone and the Dowson and Higginson equation of state^5^. Many definitions of pressure-viscosity coefficient are in current use. A few are listed below.

## Some definitions of alpha

The qualitative prediction of the EHL thickness profile is rightly regarded as one of the greatest achievements in tribology^6^ and the Hamrock and Dowson formula^7^ for central film thickness, *h*_*C*_, of a point contact is widely used to estimate the thickness of the oil film in machinery in spite of a lack of quantitative validation.

2 $$h_{c} = 1.9R^{0.464} E^{ - .073} \left( {U\mu_{0} } \right)^{0.67} F^{ - 0.067} \left( {\alpha^{*} } \right)^{0.53}$$

Notice that parameters for shear-thinning and compressibility, which have a measurable effect on film thickness, are absent from this formula.

In classical EHL a single property, the pressure-viscosity coefficient or “alpha value”, quantifies the isothermal pressure dependence of the viscosity of the liquid lubricant for prediction of film thickness. Progress toward a successful definition of this property has been hampered by the refusal of those working in classical EHL to acknowledge the existence of the accurate measurements of the piezoviscous effect available for nearly a century^2^. See, for example, a tribology textbook^8^. One definition of the pressure-viscosity coefficient, *α*_*B*_(*T*), invokes the “Barus equation” (1), requiring exponential response for some interval in pressure near ambient.

Another definition, the secant pressure-viscosity coefficient^9^, *α*_*S*_, also is related to Eq. (1).

3 $$\alpha_{S} \left( {T,p} \right) = \frac{{\ln \left( {\frac{{\mu \left( {T,p} \right)}}{{\mu \left( {T,p = 0} \right)}}} \right)}}{p}$$

This definition and another, the tangent pressure-viscosity coefficient, *α*_*T*_ require that a pressure be specified.

4 $$\alpha_{T} \left( {T,p} \right) = \frac{{d\left( {\ln \mu } \right)}}{dp}$$

The tangent pressure-viscosity coefficient, evaluated at the Hertz pressure of a concentrated contact, influences the slope of the logarithmic portion of a traction curve, a slope which has been incorrectly associated with Eyring theory. The “conventional” pressure-viscosity coefficient is^10^ *α*_0_.

5 $$\alpha_{0} \left( T \right) = \left. {\frac{{d\left( {\ln \mu } \right)}}{dp}} \right|_{p = 0}$$

The Hamrock and Dowson formula (2) employs yet another definition of pressure-viscosity coefficient, the reciprocal asymptotic isoviscous pressure coefficient, α*, a sort of weighted average of local coefficients.

6 $$\alpha^{*} = \frac{1}{{p_{isovis} \left( {p \to \infty } \right)}} \,$$

This definition was suggested by Blok^11^ considering the behavior of a mineral oil at room temperature. The isoviscous pressure is found from integration^11,12^.

7 $$p_{isovis} \left( p \right) = \int_{0}^{p} {\frac{{\mu \left( {p = 0} \right)d\tilde{p}}}{{\mu \left( {\tilde{p}} \right)}}}$$

The isoviscous pressure provides a (Weibull) transformation whereby a piezoviscous solution for the pressures in the Reynolds equation can be found from an isoviscous solution^12^. The reciprocal asymptotic value is found by setting the argument of the isoviscous pressure function to infinity.

It would seem that the integration to infinite pressure would make this definition of the pressure-viscosity coefficient untenable. However, the integration may quickly converge to a good approximation of the asymptotic isoviscous pressure for many oils within the inlet pressure range when the temperature is low.

## A problem for the Hamrock and Dowson coefficient, a*

**Figure 1:**
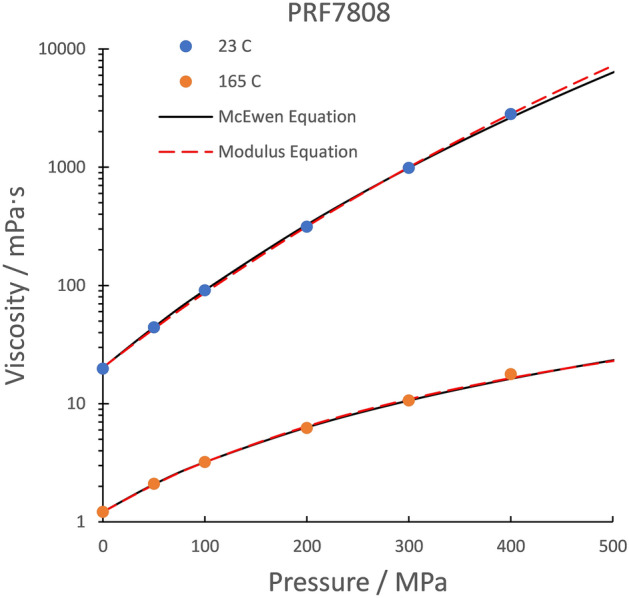
Two pressure viscosity isotherms for a jet oil comparing the McEwen and the Modulus equations. The Hamrock and Dowson pressure-viscosity coefficients are zero for the Modulus equation.

One pressing problem for EHL film thickness prediction is the film forming capability at high temperature in a roller bearing of a jet engine oil, meeting specification PRF 7808^13^. The limiting low shear viscosity, *μ*(*T*, *p*) has been measured in a falling cylinder viscometer of the type introduced by Bridgman^2^ nearly a century ago. These viscosities are listed in Table 1 for temperatures from 23 to 220 °C. The inflection in the log viscosity versus pressure curve occurs at a viscosity of 20 Pa∙s. The viscosities at 23 and 165 °C are plotted in Fig. 1 along with two correlations for the slower than exponential response at low pressures.

**Table 1:** Viscosities of a jet oil.

PRF 7808 Grade 3, Viscosities in Pa∙s					
*p* / GPa	23 °C	50 °C	100 °C	165 °C	220 °C
0.0001	0.0198	0.00843	0.00272	0.00122	0.000709
0.05	0.0442	0.01689	0.00497	0.0021	0.001371
0.1	0.0908	0.0312	0.00804	0.00321	0.00195
0.2	0.314	0.0915	0.01835	0.00622	0.00341
0.3	0.988	0.228	0.0373	0.01065	0.00547
0.4	2.82	0.536	0.0716	0.01777	0.00862
0.5	7.51	1.195			0.0122
0.6	19.52	2.55			
0.7	48.2	5.43			
0.8	120				
0.9	316				
1	808

The McEwen equation^14^ is used by many laboratories that do accurate measurements of viscosity at elevated pressures in viscometers. It is significantly better suited for the slower than exponential response than the third Roelands^15^ model often used in classical EHL and can be written with either *α*_0_ or α* as a parameter.

8 $$\mu = \mu_{0} \left( {1 + \frac{{\alpha_{0} }}{q}p} \right)^{q} = \mu_{0} \left( {1 + \frac{{\alpha^{*} }}{q - 1}p} \right)^{q}$$

The parameters of a fit to the data for pressures up to 400 MPa are listed in Table 2 along with those of the isothermal form of the Modulus equation^9^.

9 $$\mu \left( p \right) = \mu_{0} \exp \left[ {\frac{p}{{1{/}\alpha_{0} + Bp}}} \right]$$

**Table 2:** Parameters of the McEwen and the Modulus equations.

PRF 7808 Grade 3				
	23 °C		165 °C	
	McEwen eqn	Modulus eqn	McEwen eqn	Modulus eqn
*μ*_0_/mPa∙s	20.1	20.2	1.22	1.22
*α*_0_/GPa^−1^	16.5	15.4	12.06	11.53
α*/GPa^−1^	14.55	0	6.79	0
*q*	8.38	–	2.29	–
*B*	-	0.0408	–	0.167
AARD	2.1%	1.9%	1.8%	2.5%

This equation is identical to the second Roelands model^15^ and has a limiting viscosity of *μ*_0_exp(1/*B*). Although there is no real asymptotic limit to viscosity, the agreement with the data in Fig. 1 is quite satisfactory. The average absolute relative deviation, AARD, is listed in Table 2 for the two correlations and the fit to the data is excellent for both.

Surprisingly, for the Modulus equation, α* is identically zero This is due to the limiting value for viscosity which causes *p*_*isovis*_(∞) to be unbounded. The McEwen equation produces values of α* that are in reasonable agreement with those obtained from the algorithm recommended by Bair^16^.

10 $$\alpha * \approx \left[ {\frac{{\mu_{0} }}{{\alpha_{N} \mu_{N} }} + \sum\limits_{i = 1}^{N} {\frac{{\mu_{0} }}{{\alpha_{i} }}\frac{{\mu_{i} - \mu_{i - 1} }}{{\mu_{i} \mu_{i - 1} }}} } \right]^{ - 1} \quad {\text{with}}\quad \alpha_{i} = \frac{{\ln \left( {\mu_{i} {/}\mu_{i - 1} } \right)}}{{\left( {p_{i} - p_{i - 1} } \right)}}$$

Equation (10) results in α* = 14.6 and 7.6 GPa^−1^ for 23 and 165 °C, respectively. The great difference in values of α* for the McEwen and the Modulus correlations, which give equivalent fits to the data, clearly show that there is insufficient information contained in the viscosities up to pressure of 400 MPa to evaluate α*. Therefore, the evaluation of the reciprocal asymptotic isoviscous pressure coefficient, α*, requires an understanding of how the viscosity depends upon pressure for pressures very much greater than the inlet zone pressure of a contact. For example, the algorithm (10) assumes exponential response for pressures greater than the highest measured pressure, *p*_*N*_, and the McEwen Eq. (8) assumes that the slower than exponential response simply continues, whereas the Modulus Eq. (9) reaches a limiting value of viscosity.

## A problem for glass/steel EHD rigs

The contact between a glass disc and a steel ball is the working component of an EHL rig, an instrument that has become the standard for studies of EHL film thickness. In some ways, this is unfortunate because the Hertz pressure cannot exceed about 0.8 GPa and is typically no more than 0.5 GPa in most commercially available test rigs because of the strength of glass. The pressure in the inlet zone must be much less. The inlet zone where the film thickness is established ends at a pressure of 50–100 MPa according to Dench et al.^17^. A bit higher inlet pressure limit of 150 MPa is given by Spikes^18^. This means that, in many cases, the viscosity in the inlet zone may not reach sufficiently high pressure to contain the information needed to evaluate the Hamrock and Dowson pressure-viscosity coefficient, α*.

Performing the integration in Eq. (7) for the McEwen Eq. (8) yields an analytical expression for the isoviscous pressure.

11 $$p_{isovis} \left( p \right) = \frac{q}{{\alpha_{0} \left( {q - 1} \right)}} - \frac{{\left( {\alpha_{0} p + q} \right)\left( {1 + \frac{{\alpha_{0} p}}{q}} \right)^{ - q} }}{{\alpha_{0} \left( {q - 1} \right)}}$$

**Figure 2:**
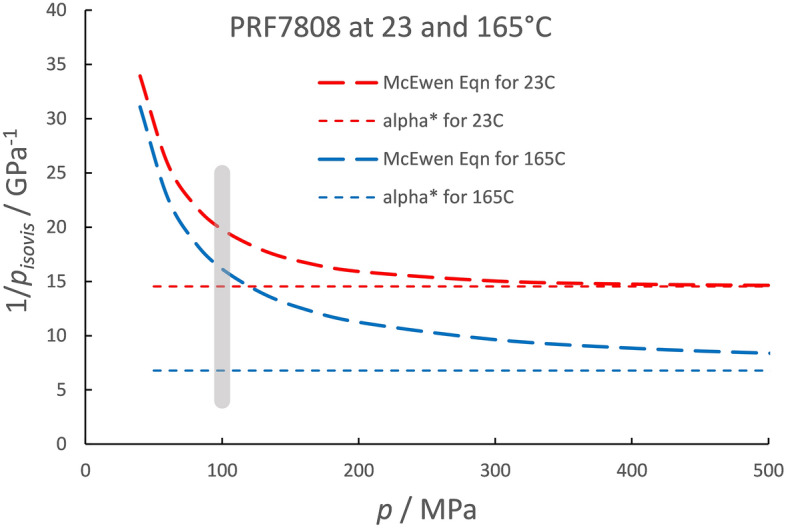
The reciprocal isoviscous pressure versus pressure given by the McEwen equation for a jet engine oil at two temperatures.

**Figure 3:**
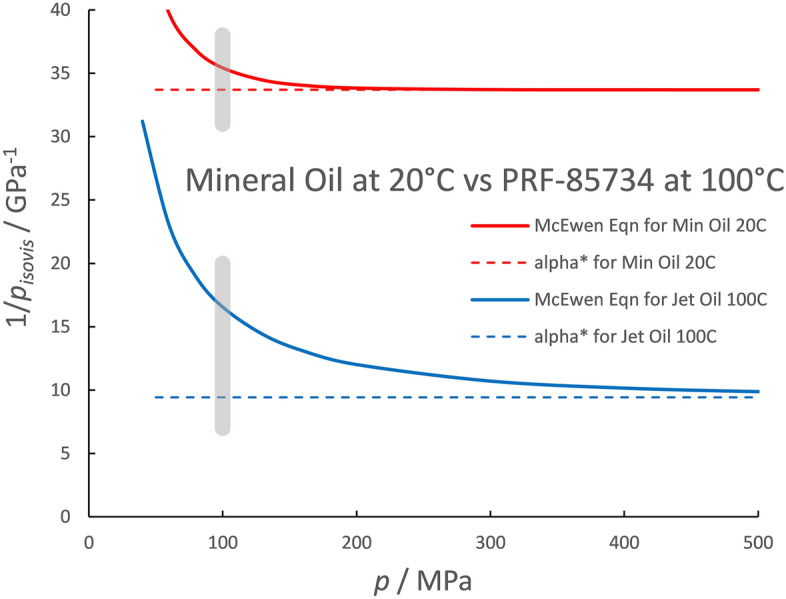
The reciprocal isoviscous pressure versus pressure given by the McEwen equation for a mineral oil at room temperature and a helicopter transmission oil at 100 °C.

The reciprocal of the isoviscous pressure is plotted in Fig. 2 for the jet engine oil meeting specification PRF 7808 at two temperatures, 23 and 165 °C. The maximum pressure of the inlet, assumed to be 100 MPa, is shown as the gray vertical line. It can be seen that, for low temperature, the reciprocal of the isoviscous pressure, 19.8 GPa^−1^, is 35% greater than the asymptotic value, α* = 14.6 GPa^−1^, at the pressure of 100 MPa. For the higher temperature, at 100 MPa the reciprocal of the isoviscous pressure is 16.2 GPa^−1^ whereas the asymptotic value is 6.8 GPa^−1^, less than half. Clearly this definition is not appropriate for a jet engine oil at temperatures above ambient. It is useful, however, for a naphthenic mineral oil at room temperature, as shown in Fig. 3 where the reciprocal of the isoviscous pressure reaches to within 5% of α* at 100 MPa. However, for a helicopter oil, meeting specification PRF-85734, at 100 °C the reciprocal of the isoviscous pressure at 100 MPa is 16.6 GPa^−1^ while the asymptotic value is 9.4 GPa^−1^.

The Hamrock and Dowson coefficient, α*, cannot be the operative pressure-viscosity coefficient in the standard glass/steel EHL rig. There is not enough information available regarding the piezoviscous effect within the inlet pressure range. The liquid cannot be responding to pressures greater than those existing in the film.

## Discussion

It has repeatedly been shown that the pressure-viscosity coefficient cannot be accurately derived from EHL film thickness measurements^19–22^; however, the method continues in use today, promoted primarily by Imperial College. The errors may be attributed to non-Newtonian inlet behavior, ignoring the temperature dependence of compressibility, operation of the EHL rig outside of the fully piezoviscous-elastic regime, and inaccurate film measurements. Another problem for the method has been shown above to be the lack of a suitable definition of the pressure-viscosity coefficient that is reported from the method. In particular, the film pressures in a glass/steel contact are insufficient to reach the asymptotic value of the isoviscous pressure. The classical Hamrock and Dowson film thickness formula, which is used in this method, requires that *p*_*isovis*_(*p*) ≈ *p*_*isovis*_(∞) within the inlet zone and, therefore, the inlet pressure would have to reach to well over 500 MPa at high temperature. The definition of pressure-viscosity coefficient and the assumed equation of state must limit the use of the classical formulas, including Hamrock and Dowson, to liquids with high Newtonian limit and to low temperature.

**Figure 4:**
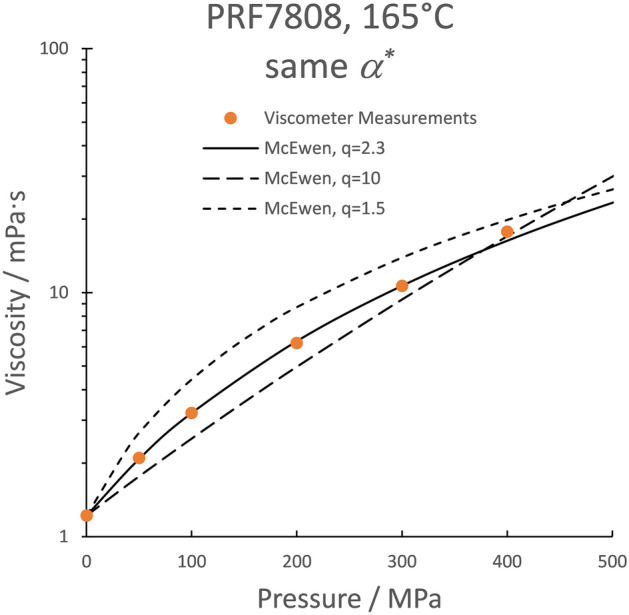
The McEwen correlation for the same value of α* = 6.8 GPa^−1^ and three values of the McEwen exponent, *q*.

The severity of the situation can be illustrated in Fig. 4 by comparing the pressure-viscosity response of the jet oil at 165 °C to that of hypothetical oils with the same value of the Hamrock and Dowson pressure-viscosity coefficient, α*, but with different values of the McEwen exponent, *q*. For the jet oil, *q* = 2.3. Values of *q* for typical lubricating oils are in the range of 1.2–20. For traction fluids *q* may exceed 100. In Fig. 3, the hypothetical oils have *q* = 1.5 and 10 with the same α* = 6.8 GPa^−1^ as the jet oil. If the inlet pressure is less than 300 MPa, the jet oil will clearly have a smaller film thickness than the hypothetical *q* = 1.5 oil given the same compressibility and Newtonian response although they have equal α*. The conventional pressure viscosity coefficients are *α*_0_ = 20, 12.1, and 7.5 GPa^−1^ for the hypothetical *q* = 1.5 oil, the jet oil, and the hypothetical *q* = 10 oil, respectively. However, it has already been shown that the conventional pressure viscosity coefficient does not correctly rank the film forming capability^23^. Two oils with the same *α*_0_ and *μ*_0_ can generate very different film thicknesses in both experiments and simulations^23^.

Fifteen years ago, this author suggested an alternative definition of pressure-viscosity coefficient, which in simulations appeared to offer a suitable replacement for α*^23^ and provides a set of curves more compact at low pressures than does α* in Fig. 4 . However, this definition also invoked the integral of Eq. (7) requiring viscosity information at pressures which may exceed the contact pressure.

## Conclusion

The Hamrock and Dowson pressure-viscosity coefficient at high temperature requires knowledge of the piezoviscous response at pressures which exceed the inlet pressure and may exceed the Hertz pressure. The definition of pressure-viscosity coefficient and the assumed equation of state must limit the use of the classical formulas, including Hamrock and Dowson, to liquids with high Newtonian limit and to low temperature. Given that this problem has existed for at least fifty years without resolution, it is reasonable to conclude that there is no suitable definition of pressure-viscosity coefficient that will quantify the piezoviscous response for an analytical calculation of EHL film thickness for temperatures above ambient.

### Consent to participate

The author consents to participate.

### Consent for publication

The author consents to publication.

## Data Availability

Will make all data available on request.

## Abbreviations

*B*: Parameter in the Modulus equation, Pa
*E*: Composite Young’s modulus, Pa
*F*: External applied load, N
*h*_*c*_: Central film thickness, m
*p*: Pressure, Pa
*p*_*isovis*_: Isoviscous pressure, Pa
*q*: McEwen exponent
*T*: Temperature, K
*U*: Mean entrainment speed, m/s
*α*_*B*_: Barus pressure-viscosity coefficient, Pa^−1^
*α*_*S*_: Secant pressure-viscosity coefficient, Pa^−1^
*α*_*T*_: Tangent pressure-viscosity coefficient, Pa^−1^
*α*_0_: Conventional pressure-viscosity coefficient, Pa^−1^
*α**: Reciprocal asymptotic isoviscous pressure coefficient, Pa^−1^
*μ*: Limiting low-shear viscosity, Pa s
*μ*_0_: Low-shear viscosity at *p* = 0, Pa s
